# Ablating all three retinoblastoma family members in mouse lung leads to neuroendocrine tumor formation

**DOI:** 10.18632/oncotarget.13875

**Published:** 2016-12-10

**Authors:** Sara Lázaro, Miriam Pérez-Crespo, Ana Belén Enguita, Pilar Hernández, Jesús Martínez-Palacio, Marta Oteo, Julien Sage, Jesús M. Paramio, Mirentxu Santos

**Affiliations:** ^1^ Molecular Oncology Unit Institute of Biomedical Investigation University Hospital “12 de Octubre”, Madrid, Spain; ^2^ Pathology Department Institute of Biomedical Investigation University Hospital “12 de Octubre”, Madrid, Spain; ^3^ Biomedical Applications and Pharmacokinetics CIEMAT (ed 70A), Madrid, Spain; ^4^ Department of Pediatrics, Stanford University, Stanford, USA; ^5^ Department of Genetics. Stanford University, CCSR Rm. 1215a. Stanford, California, USA; ^6^ Molecular Oncology, Institute of Biomedical Investigation University Hospital “12 de Octubre”, Madrid, Spain; ^7^ Centro de Investigaciones Biomédicas en Red de Cáncer (CIBERONC), Madrid, Spain

**Keywords:** retinoblastoma family, lung cancer, urethane, DHPN

## Abstract

Lung cancer is a deadly disease with increasing cases diagnosed worldwide and still a very poor prognosis. While mutations in the retinoblastoma (*RB1*) tumor suppressor have been reported in lung cancer, mainly in small cell lung carcinoma, the tumor suppressive role of its relatives p107 and p130 is still a matter of debate. To begin to investigate the role of these two Rb family proteins in lung tumorigenesis, we have generated a conditional triple knockout mouse model (TKO) in which the three Rb family members can be inactivated in adult mice. We found that ablation of all three family members in the lung of mice induces tumorlets, benign neuroendocrine tumors that are remarkably similar to their human counterparts. Upon chemical carcinogenesis, DHPN and urethane accelerate tumor development; the TKO model displays increased sensitivity to DHPN, and urethane increases malignancy of tumors. All the tumors developing in TKO mice (spontaneous and chemically induced) have neuroendocrine features but do not progress to fully malignant tumors. Thus, loss of Rb and its family members confers partial tumor susceptibility in neuroendocrine lineages in the lungs of mice. Our data also imply the requirement of other oncogenic signaling pathways to achieve full transformation in neuroendocrine lung lesions mutant for the Rb family.

## INTRODUCTION

Lung cancer is a major health problem worldwide, as it belongs to the most deadly cancer condition with overall poor prognosis and only a small percentage surviving more than 5 years. Much more effort is needed for understanding the molecular mechanisms underlying the disease and for the development of early detection techniques and novel effective therapies.

Approximately 15–20% of lung tumors show characteristics of neuroendocrine cells. Pulmonary neuroendocrine (NE) tumors include, depending on the tumor size and the number of mitoses, a spectrum of tumors that range from the low-grade, typical carcinoid (TC), through intermediate-grade, atypical carcinoid (AC) to the high-grade, large-cell neuroendocrine carcinoma (LCNEC) and small-cell lung carcinoma (SCLC). The benign NE tumors that precede the carcinoid tumors are usually small nodular NE proliferations called tumorlets [1–3]. Tumorlets are composed of aggregates of NE cells that demonstrate morphology similar to those of carcinoid tumors. Carcinoid tumors (TC and AC) (account for 1% to 2% of lung malignancies), show similar characteristic histologic patterns and the differential diagnoses is based on mitotic index and proliferative status (2 mitosis per 10 high-power field and 5% Ki-67 positive staining for TC, and 2–10 mitosis per 10 high-power field and 5%–20% Ki-67 positive staining or the presence of necrosis for AC). Prognosis and survival rates for AC are significantly reduced compared to that for TC [1, 2]. For patients, best management is surgical resection as no proven optimal therapy has been demonstrated for these cases.

The RB family of proteins, composed by pRb and its relatives p107 and p130 (encoded by the genes *RB1*, *RBL1* and *RBL2* respectively), plays a crucial role in the control of cell cycle progression through its ability to bind the E2F transcription factors in a complex regulatory network. RB family also participates in multiple cellular processes beyond cell cycle progression. Observations in various human cancers in which genetic inactivation of *RB1* has been reported, and the analysis of genetically engineered mice have identified pRb as a major tumor suppressor. The *RB1* gene is inactivated in most cases of specific cancer types such as retinoblastoma, small cell lung cancer (SCLC) and osteosarcoma, whereas alterations of its family members p107 and/or p130 is still a matter of debate, being rarely mutated in human tumors [4–6].

Several studies have shown that p107 as well as p130 may function as tumor suppressors in the context of lung carcinogenesis [7–9] as well as in other tissues [10, 11], including our own studies in mouse bladder and epidermal carcinogenesis [12–15].

Mouse models have been developed to mimic *in vivo* human lung cancer including chemical carcinogenesis approaches such as DHPN [N-bis(2-hydroxypropyl) nitrosamine) or urethane. DHPN, a potent mutagen and a wide-spectrum carcinogen in rodents induces lung tumors when given in drinking water [16–18]. Mutational activation of Kras has been described in these rodent lung tumors [19, 20]. Urethane (ethyl carbamate), a component of various food products and cigarette smoke, induces the formation of lung tumors in animal models. The mechanism involves the formation of DNA adducts which causes extensive damage in lung cells leading to tumor formation [21]. The presence of Kras Q61R mutations have been also frequently observed in mouse urethane-induced tumors [22].

In an attempt to contribute to defining the roles of the retinoblastoma family in lung tumorigenesis we have generated a new mouse model based on the ablation of all three retinoblastoma family members (*Rb^F/F^; p130^F/F^;p107^−/−^*) in pulmonary cells; hereafter referred to as ”triple knock out” mice, TKO mice. We have also examined its susceptibility to DHPN or urethane. Our results show that 1) inactivation of multiple pocket protein in lung is not sufficient to induce malignant lung cancer but is sufficient to render tumorlets; 2) DHPN contributes specifically to the development of low-grade neuroendocrine tumors and 3) urethane increases malignancy of tumors that do not reach higher grades of malignancy, indicating that other signaling pathways must be involved in the contribution to late stages in lung neuroendocrine tumorigenesis.

## RESULTS

### Rb and p130 ablation in adult p107−/− lungs leads to the spontaneous development of tumorlets

Inactivating all three Rb family pocket proteins in the lung was achieved by inoculating Ad5-CMVcre virus to *Rb^F/F^; p130^F/F^;p107^−/−^* adult mice [15] by intranasal or intratracheal administration in doses ranging from 5 × 10^8^ pfu to 10^10^ pfu. A wide variety of pulmonary epithelial cells is uniformly targeted in the lung as seen by lacZ staining 6 days after infection of ROSA 26R [23] adult mice (Supplementary Figure S1A–S1D) and corroborated by the presence of cre 4 days after infection (Supplementary Figure S1E–S1G).

We observed a significant reduction of *Rb1* and *Rbl2* gene expression in the lungs of infected mice (*n* = 27), but not in control mice (noninfected littermates *n* = 20) (Supplementary Figure S2A, S2B). This reduction was more evident in the 3 isolated tumorlets analyzed (Supplementary Figure S2C, S2D). Likewise, pRb and p130 proteins were detected by immunohistochemical stainings in normal lung epithelia/alveoli (Supplementary Figure S2G, S2H), but not in the tumorlets observed (Supplementary Figure S2E, S2F). These data confirm that gene recombination is achieved in the lungs of infected mice and occurs in spontaneous tumorlets.

**Figure 1:**
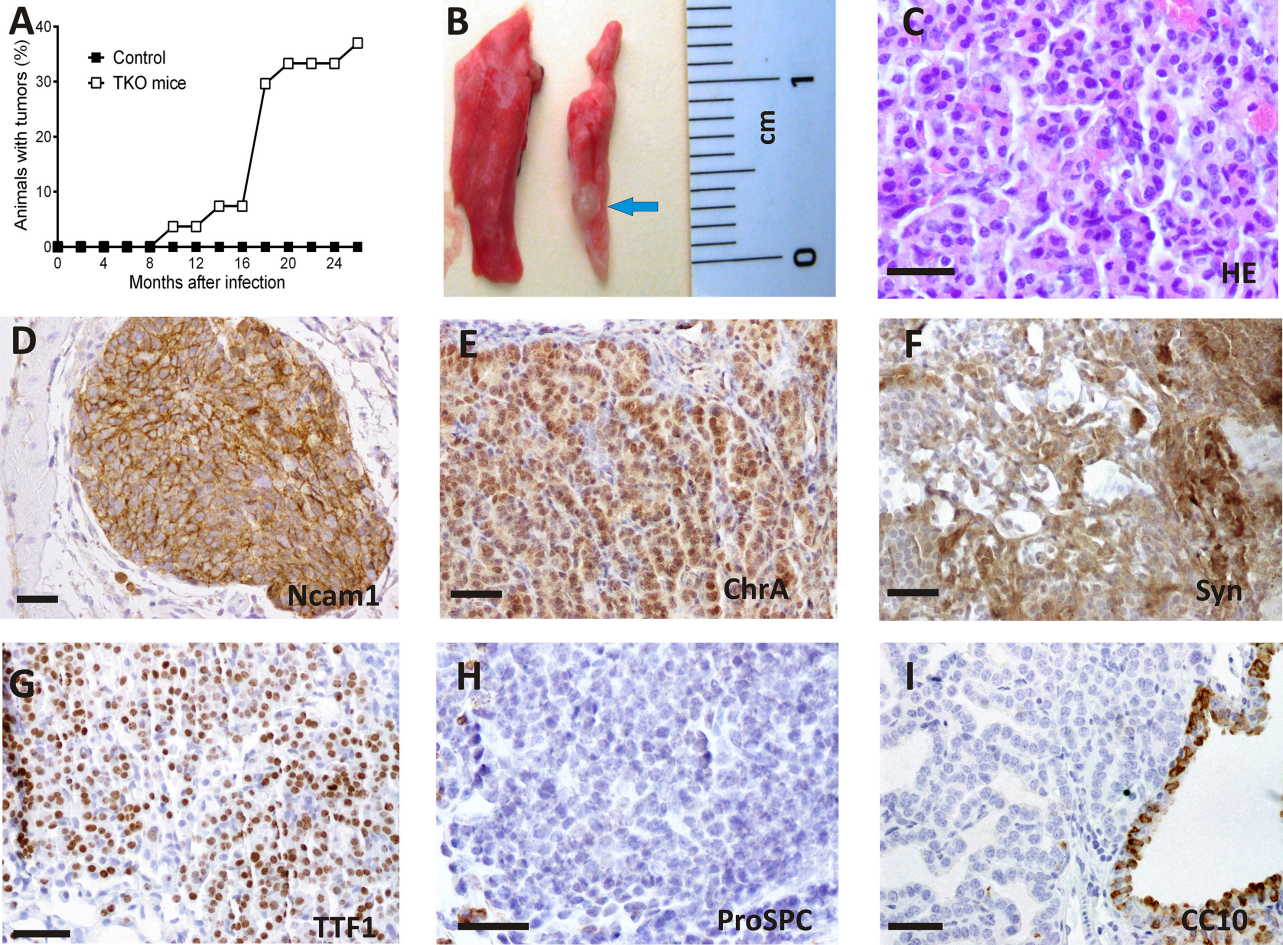
Spontaneous tumorlet development after ablation of Rb family members in adult TKO lungs (**A**) Tumor latency of TKO mice. (**B**) Example of an external view of a tumorlet (arrow) in the lung 17 months after cre delivery. (**C**) Histology of a tumorlet composed of a uniform population of cells. (**D**–**I**). Immunohistochemical staining for the quoted proteins. Representative images are shown. Positive immunostaining for N-cam1 (D), ChrA (E) ,Syn(F) and TTF-1 (G), and negative for proSPC (H) and CC-10 (I). HE hematoxylin and eosin stain. Bars = 50 μm

The histologic analysis of the infected lungs revealed the presence of lesions that displayed features of tumorlets. After periods of 9–24 months post infection, 37% (10 out of 27) of animals infected with Ad5-CMVcre virus developed tumorlets (Figure 1A). These lesions were occasionally visually detectable (Figure 1B). Progression to malignant tumors was not observed even for periods of 24 months after cre delivery. No differences were observed in the phenotype, period of latency or tumor burden regarding the doses of virus used in the TKO mice.

These tumorlets consisted of nodular aggregates of proliferating neuroendocrine tumor cells with uniform morphology (Figure 1C), average size 1,035 ± 0,262 mm^2^ ( Mean ± SEM) with parenchymal and bronchiolar location in the adult lung (Table 1) and striking morphological and immunophenotypical similarities to human counterparts (Supplementary Figure S3) [1, 2, 24]. Indeed, Immunohistochemical analysis of the mouse tumorlets showed (Figure 1C–1I) positive staining for neuroendocrine markers such as neural cell adhesion molecule (NCAM1), Chromogranin (CHR A), synaptophysin (SYN), and thyroid transcription factor-1 (TTF-1) and negative staining for pro-surfactant protein C (pro-SPC) and Clara cell specific protein (CCSP/CC-10), two markers of lung alveolar and bronchiolar epithelial cells, respectively. Positive Staining for cytokeratin CK8 and absence of cytokeratin CK5 was observed (not shown).

**Table 1:** Number, localization and size of spontaneous tumorlets

Animal	Number of tumors/mice	Localization	Size (mm^2^)
A	1	parenchymal	0,267
B	2	parenchymal	0,505
		tracheal	0,287
C	4	parenchymal	1,47
		bronchiolar	0,053
		bronchiolar	0,033
		parenchymal	0,315
D	2	parenchymal	2,336
		parenchymal	0,167
E	2	parenchymal	0,272
		parenchymal	0,627
F	1	parenchymal	1,656
G	1	bronchiolar	2,153
H	1	parenchymal	1,663
I	1	parenchymal	3,711
J	1	parenchymal	1,047

The development of spontaneous tumorlets shows that disruption of all three pocket proteins drives development of neuroendocrine benign lesions but is not sufficient for the progression of these spontaneous tumors to a malignant phenotype.

### Molecular characterization of spontaneous tumorlets and resemblance to humans

**Figure 2:**
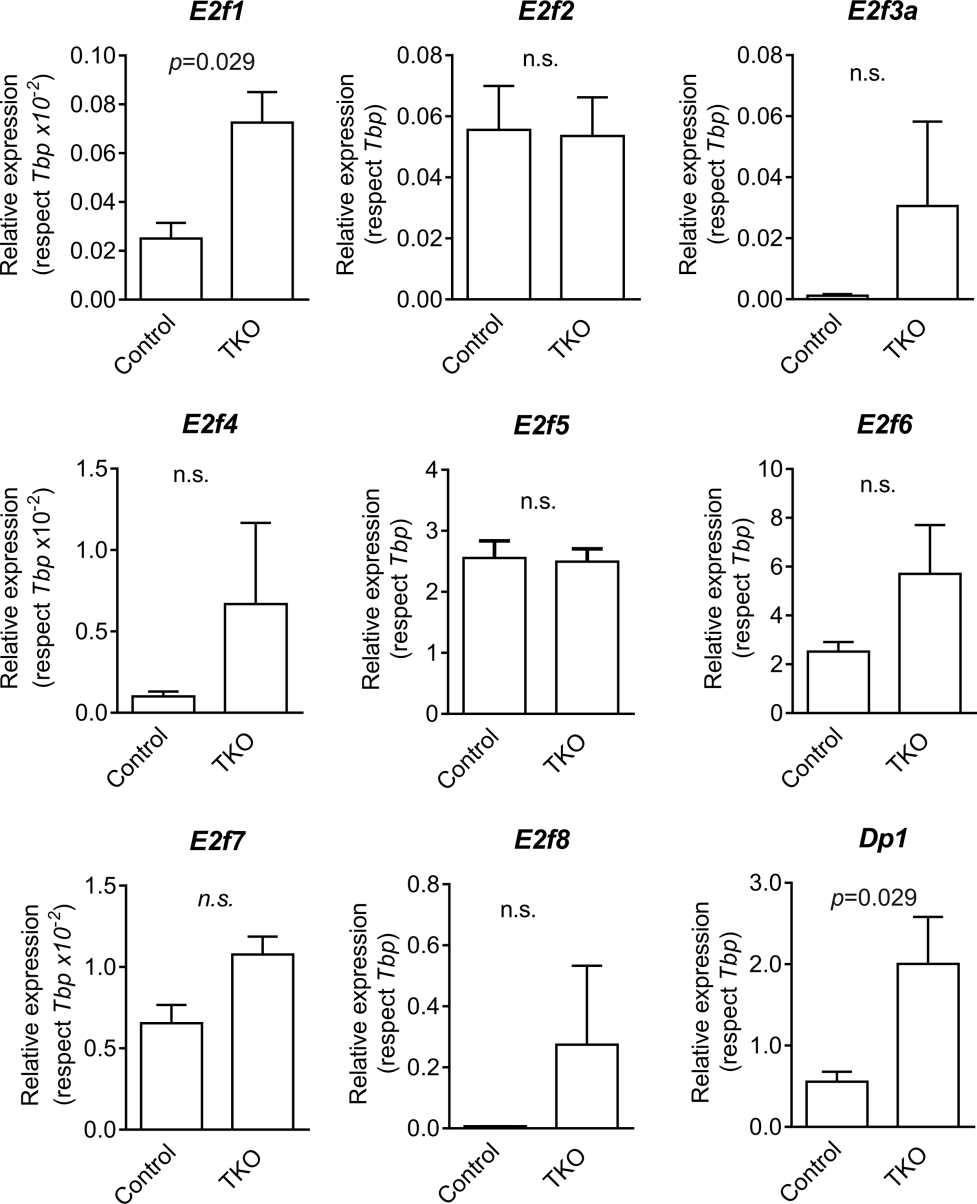
Expression of E2f transcription factors genes in mouse lungs Expression of *E2f1, E2f2, E2f3a, E2f4, E2f5, E2f6, E2f7, E2f8* and *Dp1* genes in control (uninfected mice, lung samples) and TKO (infected mice, lung samples) were monitored by RT-qPCR (respect to Tbp). *p* values were obtained by the Mann Whitney test (n.s: not significant; *n* = 4).

**Figure 3:**
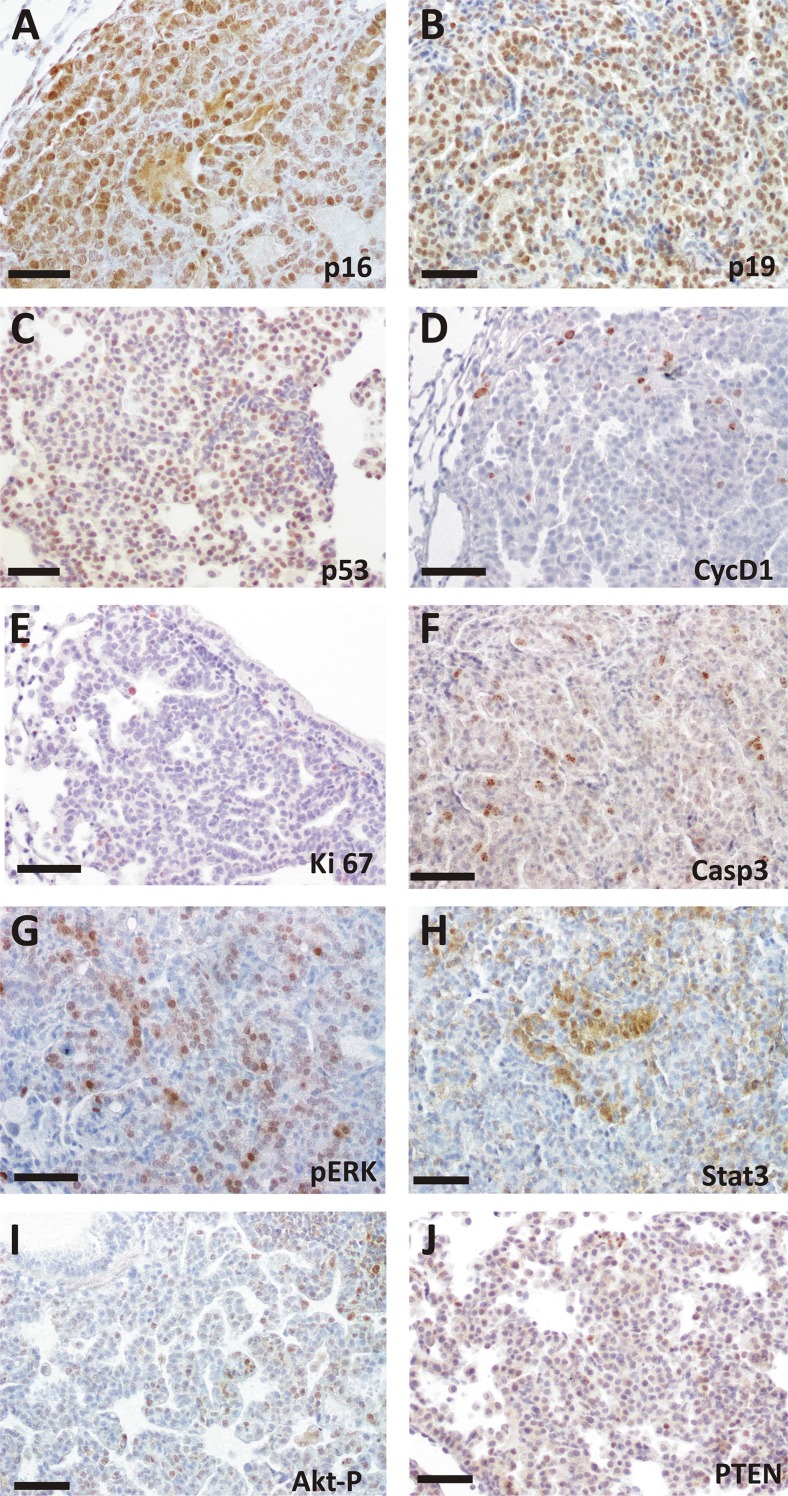
Immunohistochemical analysis of spontaneous tumorlets in TKO mice Representative images from at least 4 different mice of immunohistochemical staining for the quoted proteins. Mouse tumorlets display expression of p16 (**A**), p19 (**B**), p53(**C**), CYCD1 (**D**). Immunostaining for Ki-67 (**E**), CASP3 (**F**), pERK (**G**), STAT-3 (**H**), AKT-P (**I**),and PTEN (**J**) is also shown. Bars = 50 μm.

Rb pocket proteins control cell cycle progression in part through binding and inactivation of the E2F family of transcription factors. We observed increased expression of the transcription factors *E2f1* and its dimerization partner *Dp1* in Ad5-CMVcre infected lungs by RT-qPCR analyses (Figure 2).The mouse tumorlets were characterized by the generalized expression of p16 and p19ARF, whereas p53 and to a lower extent CyclinD1 displayed scattered expression (Figure 3A–3D). Cleaved Caspase 3 was detected in Rb family ablated tumorlets indicating some induction of apoptosis (Figure 3F).

In addition, we observed activation of ERK signaling, as demonstrated by immunohistochemical analysis using phospho-specific antibody, and STAT3 activation as shown by the specific STAT3 nuclear localization (Figure 3G–3H). In contrast, tumors did not show increased AKT activity possibly due to PTEN expression (Figure 3I–3J).

The immunophenotypic characterization of human tumorlets was also performed in 30 tumors and many similarities with the mouse model were observed. The positive staining for the neuroendocrine markers CD56, CHR, SYN and TTF-1 is shown in Supplementary Figure S3. As reported for the animal model (Figure 2), human tumorlets are characterized by expression of p16 and high levels of PTEN (Supplementary Figure S3). Thus, the tumors that develop in the animal model share multiple histopathological and immunohistochemical features with their human counterparts.

### Chemical carcinogenesis in the TKO lung model

Given that the ablation of the three Rb family members only gives rise to benign spontaneous tumorlets, we sought to evaluate whether this alteration confers susceptibility to chemical carcinogenesis. We took advantage of the carcinogens DHPN and urethane, two chemical compounds derived from nitrosamines and ethyl carbamate, respectively, known inducers of lung tumors in mice [16, 25].

### DHPN favors TC development in Rb family deleted lungs

**Figure 4:**
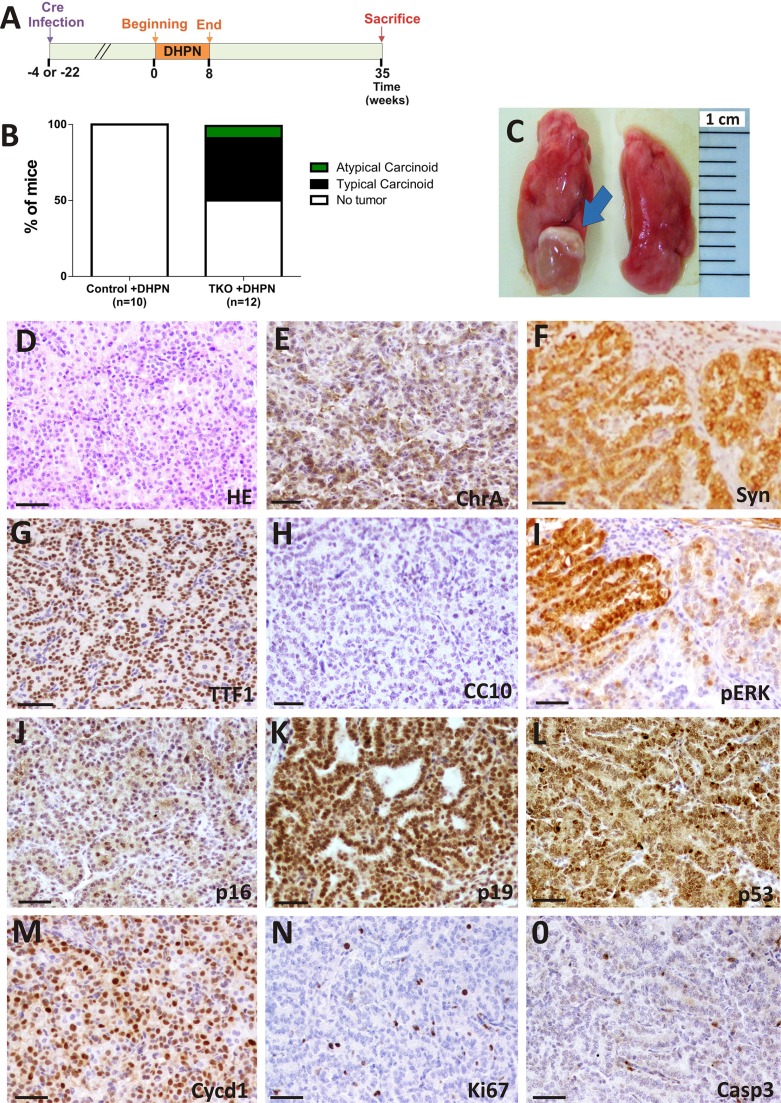
DHPN carcinogenesis in TKO mice Rb family-deficient mice are more susceptible to DHPN-induced lung tumorigenesis than control mice. (**A**) Experimental schedule for DHPN administration to control and TKO mice. (**B**) Incidence and histological spectrum of lesions in control and TKO mice treated with DHPN (Control *n* = 10, TKO *n* = 12). (**C**) Example of external view of a typical carcinoid (arrow). Representative photographs from the lungs of TKO mice. Representative histology (*n* ≥ 5) (**D**) and immunohistochemical characterization (**E**–**O**) of typical carcinoids arisen in DHPN treated TKO mice. Tumors show positive staining for the neuroendocrine markers CHR (E), SYN (F), TTF-1(G) and negative for CC10 (H). The presence of ERK 1/2 P (I, serial section of F), p16 (J), p19 (K), p53 (L) Cycline D1 (M), Ki-67 (N), Caspase 3 (O) is shown. Bars = 50 μm.

DHPN was given to mice at a concentration of 0.1% in drinking water for 8 weeks to uninfected (group referred to as control+DHPN *n* = 10) or infected mice (TKO+DHPN group *n* = 12). In this case, the beginning of DHPN administration was done at two different time points: 4 or 22 weeks after Ad5-CMVcre delivery. No differences were observed between these two subgroups. Mice were sacrificed 35 weeks after starting DHPN treatment (Figure 4A).

The administration of DHPN for 8 weeks was unable to induce tumor formation in uninfected mice (*n* = 10). In contrast, when DHPN was administered after Ad5-CMVcre infection, six mice out of twelve developed lung tumors (Figure 4B). Lung lesions were macroscopically visible (Figure 4C) and histologically diagnosed as typical carcinoids (TC) (Figure 4D), except for one case diagnosed as atypical carcinoid (AC). This differential diagnosis is based, as in human patients, by their relative mitotic and proliferation index (2 mitosis per 10 high-power field for TC and Ki-67 ≤ 5%). 20 tumors were analyzed and immunohistochemical analyses showed homogeneous positive staining for NE markers CHR A, SYN and TTF-1 and negative for CC-10, a marker of bronchiolar epithelial cells (Figure 4E, 4F, 4G and 4H respectively). We did not observe a statistically significant increase in the proliferation index of non-neoplastic lung epithelium between the TKO and control mice upon DHPN treatment (TKO+DHPN Ki-67 = 2,976 ± 0,25 SEM, control+DHPN Ki-67 = 2,181 ± 0,3939 SEM *p* = 0,1981), indicating that the differences in tumorigenesis observed upon DHPN treatment are not mediated by generalized increase in proliferation due to Rb family loss in non tumoral areas.

The DHPN-induced tumors showed, in addition to Ki-67 staining (Figure 4N), signs of apoptosis, as indicated by cleaved caspase 3 detection (Figure 4O). We also observed a generalized expression of p16 (Figure 4J), p19 (Figure 4K), p53 (Figure 4L) and CyclinD1 (Figure 4M) in these tumors. Active ERK was observed in scattered areas of the tumors (Figure 4I), similarly to spontaneous tumorlets (Figure 3G). No tumors were developed in control mice, which is unexpected, given the reported activation of Kras in DHPN-induced mouse lung tumors [20].

These data indicate that the loss of Rb family confers sensitivity to DHPN-induced lung tumor development, favoring specifically the occurrence of lung typical carcinoids (TC).

### Urethane treatment in the TKO model: increase in AC tumors

**Figure 5:**
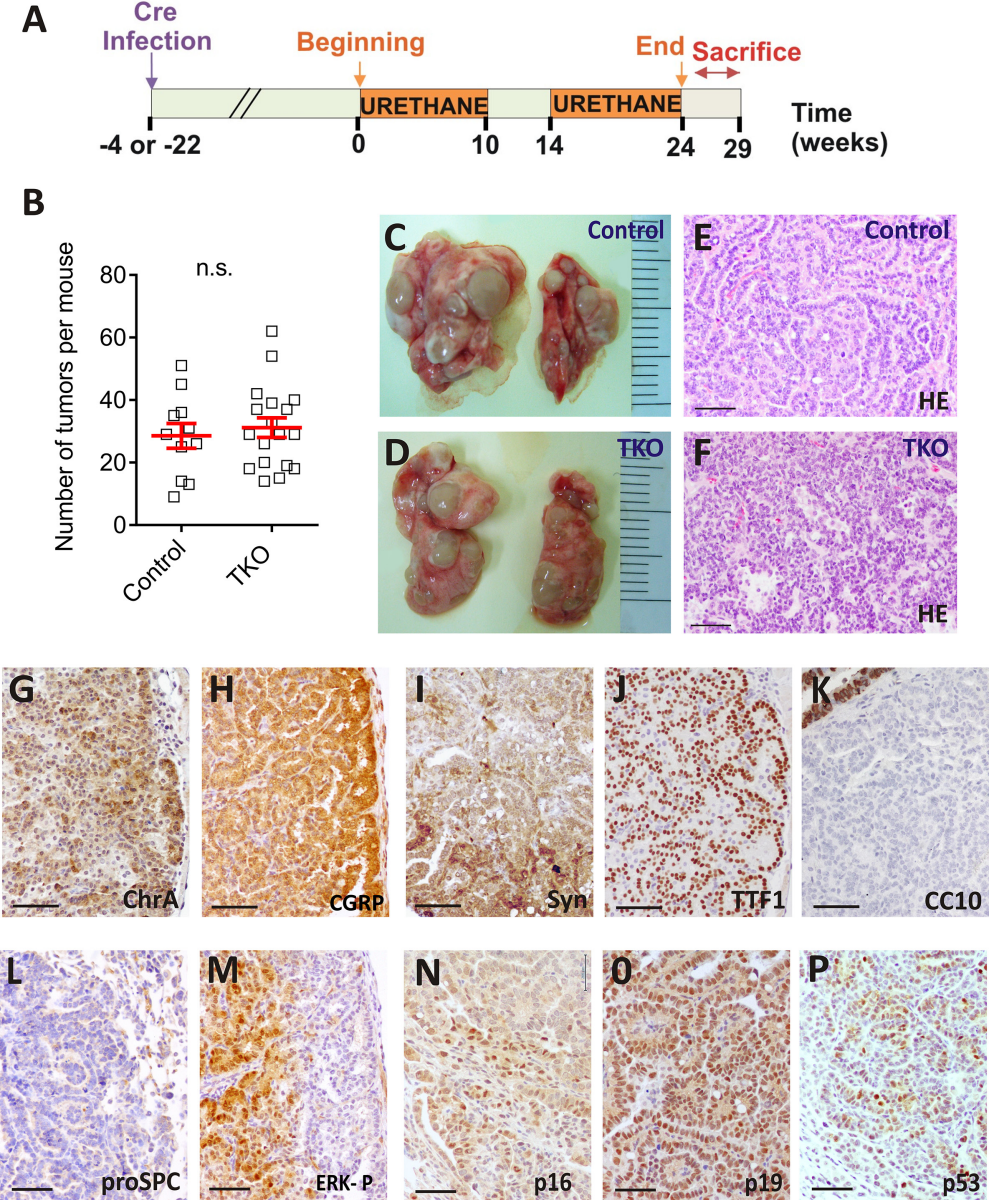
Urethane induces lung carcinogenesis in TKO mice (**A**) Experimental protocol for urethane administration to control and TKO mice. (**B**) Lung tumor multiplicities in the urethane treated control and TKO. (Student's unpaired *t*-test; control *n* = 11, TKO *n* = 18. n.s.= not significant) (**C**–**D**) Lung and tumor appearance in urethane treated, control (C) and TKO (D) mice. (**E**–**F**) Hematoxylin and eosin staining of atypical carcinoid tumors from urethane treated, control (E) and TKO (F) mice. (**G**–**P**) Representative (*n* ≥ 5) immunohistochemical analyses of CHR A (G), CGRP (H), SYN (I), TTF-1(J) CC10 (K), pro SPC (L), ERK 1/2 P (L, serial section of H), p16 (N), p19 (O), p53 (P) in lung tumors from urethane treated mice. The photographs are from Ad5-CMVcre infected mice. Bars = 50 μm

Urethane, a well characterized lung carcinogen in rodents, which also produces Kras activation [22] was administered to uninfected control littermates (Control+urethane group *n* = 11) or infected mice (TKO+urethane group *n* = 18). For the adenovirus infected mice urethane administration began at two different time points: 4 or 22 weeks after Ad5-CMVcre delivery (Figure 5A). We thus monitored whether Rb family ablation can also favor urethane carcinogenesis. In contrast with the DHPN carcinogenesis, all treated mice developed tumors, being exclusively of neuroendocrine type irrespective to Cre recombination. No differences were observed regarding the time of infection and the starting point of the carcinogen administration. Mice were sacrificed 24 to 29 weeks after the beginning of urethane treatment. A number of *n* = 55 and *n* = 67 tumors were analyzed for the control+urethane and the TKO+urethane groups respectively.

The tumor multiplicity induced by urethane was similar in the TKO and uninfected littermates (Figure 5B). Lung tumors were visible (Figure 5C, 5D) and classified by histological examination mainly as typical carcinoid (TC) and atypical carcinoid (AC). Occasionally, high grade, malignant tumor type large cell neuroendocrine lung cancer (LCNEC) arose. In all cases tumors expressed NE differentiation markers, being negative for specific alveolar and bronchiolar epithelial cells antibodies (Figure 5G–5L).

**Figure 6:**
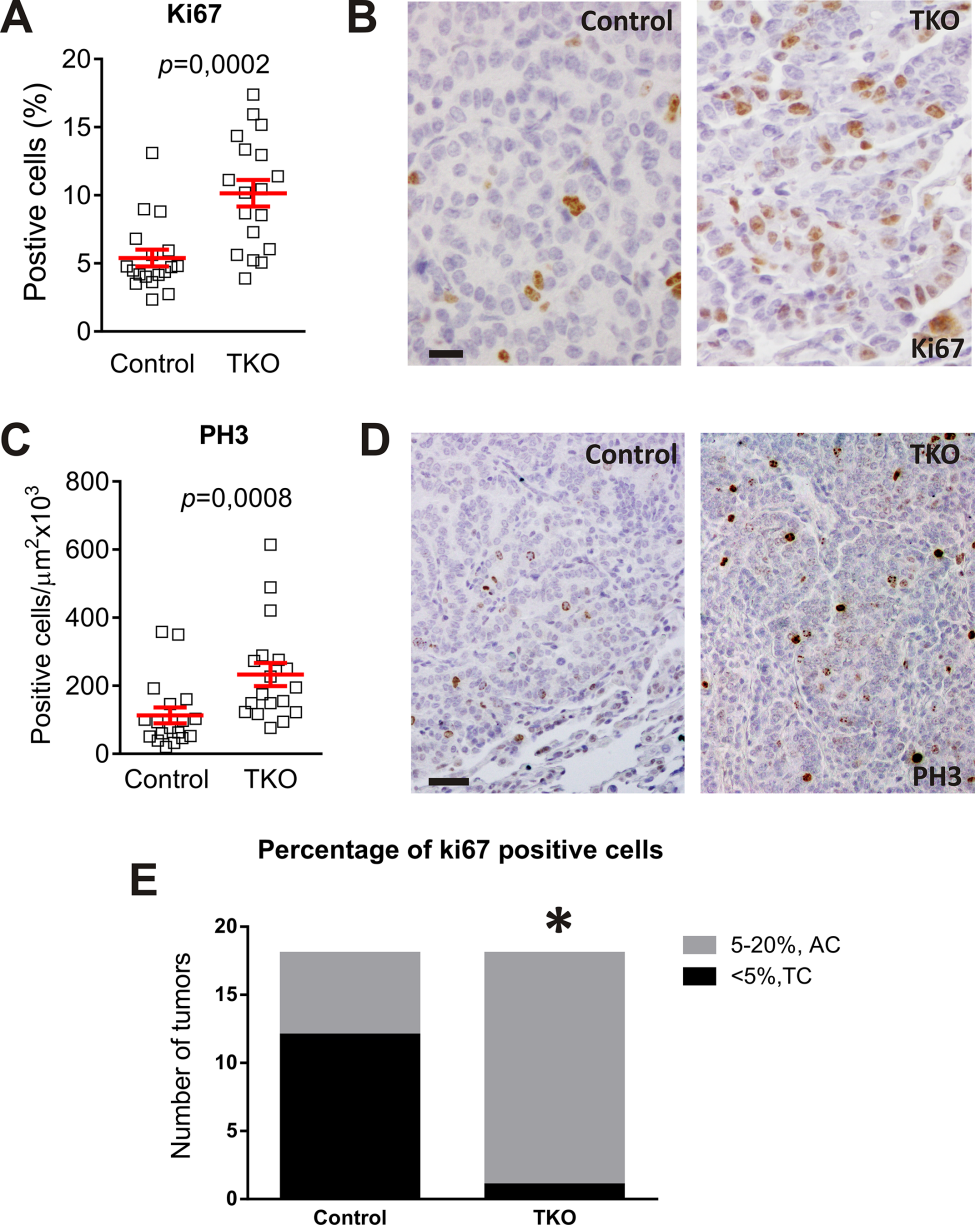
Urethane favors the development of Atypical Carcinoids in Rb family deleted lungs Analysis of proliferation in urethane treated mice control and TKO tumors of similar size by immunostaining for Ki-67. (**A**) Quantification. *p* values were obtained by the Mann Whitney test (*p* < 0,001). (**B**) Representative (*n* ≥ 5) immunostaining. Bars = 20 μm. Analysis of proliferation in urethane treated mice infected and non-infected tumors of similar size by immunostaining for PH3. (**C**) Quantification. *p* values were obtained by the Mann Whitney test (*p* < 0,001) (**D**) Representative immunostaining. Bars = 50 μm (**E**) Refinement of diagnosis after quantification of proliferating cells.*Fischer´s exac*t* test *p* = 0.0003, *n* = 18. AC: Atypical Carcinoids. TC: Typical Carcinoids.

However, the increased proliferation (determined by 5–20% cells showing Ki-67 positive staining; Figure 6A, 6B) [1, 2] and augmented mitotic index (monitored by positive phosphorylated histone H3 staining; Figure 6C, 6D) [26], indicated that the percentage of the intermediate grade carcinoid tumor, AC, is significantly higher in TKO mice (Figure 6E). Those cases in which necrosis was observed the proliferating index was also > 5%, accounting for AC. As in the case of DHPN carcinogenesis, no differences were found in the proliferation of the non tumoral tissue between control and TKO groups upon urethane treatment (TKO+urethane Ki-67 = 3,229 ± 0,2149 SEM, control+urethane Ki-67 = 2,693 ± 0,25 SEM *p* = 0,214).

We then monitored the expression of pRb and p130 by immunohistochemistry (Supplementary Figure S4B). Only a scattered expression of p130 mostly localized in cells surrounding the tumor is detected in the control urethane-induced tumors (Supplementary Figure S4B). After urethane administration no differences were observed in the expression of *E2f* gene family between control and TKO lung tumors (Supplementary Figure S4A). Furthermore, we evaluated the status of different molecular events after urethane exposure. The presence of p16, p19 and p53 is observed in the tumor cells, both infected and uninfected with adeno-cre virus (Figure 5N, 5O, and 5P, respectively). ERK-P was also detected in all urethane induced tumors confirming the activation of the Ras pathway (Figure 5M).

Our data suggest that urethane itself does no exert strong promoting effects on lung carcinogenesis after deletion of Rb family members. In order to maintain identical genetic background, all the animals used in this study (infected, uninfected, treated or not with the carcinogen) are null for the p107 allele. This fact and the reported decrease of *Rb* in urethane induced lung tumors [27] may explain that the differences observed in the expression of the E2Fs transcription factors and downstream targets are attributable to urethane treatment rather than to the ablation of the three Rb family genes. In this context, the loss of p130 alters the proliferation of induced tumors that favors a higher grade of malignancy without affecting the tumor multiplicity, tumor burden or accelerating the onset of the tumors after carcinogen treatment.

## DISCUSSION

The roles of pRb as a tumor suppressor in lung cancer have been widely reported [28]. Here we show that ablation of the three members of the retinoblastoma family conducted by Ad5-CMVcre, which targets uniformly a wide variety of adult lung epithelial cells, leads to spontaneous development of tumorlets, benign precancerous NE lesions. The adenoviral cre delivery to the lungs has proven to be a very efficient strategy to develop lung tumors in genetically engineered mouse models [29–31].

The exclusive appearance of neuroendocrine tumors is in agreement with the reported consequences of the Rb loss in distal and conducting airway epithelial cells of the lungs leading to an expansion of NE cells [7, 8, 32]. In addition, RB loss is observed in > 90% of human SCLC [33, 34] and a mechanism for transformation of adenocarcinomas to SCLC by *RB1* inactivation has been suggested [35]. Moreover, evidences of NE differentiation in tumors with loss of *Rb1* have been reported for other tumor types such as pituitary and thyroid tumors [36], adrenal glands [28] and prostate [37, 38].

However, we observed that the precancerous tumorlets never progressed into higher grade of malignancy. This finding is in contrast with the reported NSCLC development observed in mice in which *Rb1* ablation is targeted to alveolar type II cells in a p107-null background [8]. Moreover, we have observed that p107 ablation is sufficient to promote tumor development in epidermis lacking pRb [13, 14]. It is tempting to speculate that the differences could be attributed to the different cell type targeted. Although our data showing the Cre expression and efficient recombination in a wide variety of lung cell types (Supplementary Figure S1) argues against this possibility, it is possible that the use of inducible Cre transgenes can cause larger recombination frequencies than the adenovirus infection, and thus increasing the possibility of malignant progression of recombined cells.

Another possibility would be related to potential oncogenic roles played by p130 in lung and thus, its elimination could preclude tumor progression. This alternative, besides being counterintuitive, is not supported by the actual data obtained from cancer mouse models. Indeed the loss of p130 in Rb-deficient cells promotes the development of neuroendocrine lesions in the lungs of mice [10], deletion of Rb and p130 in lung epithelial cells leads to hypercellularity due to defective apoptosis [8]; and p130 acts as a tumor suppressor in the context of Rb and p53 loss (rendering SCLC [9] or NSCLC [30] depending on the targeted cell compartment) or Kras activation (rendering NSCLC [39]). Moreover, using the same mouse model and the same adenoviral vector the development of overt tumors in mouse bladder [15] or liver [40] have been previously reported, and the ablation of retinoblastoma proteins in epidermis using a K14creER promoter to express Cre recombinase also caused spontaneous tumor development in surviving mice [Bornachea O PhD thesis https://repositorio.uam.es/handle/10486/667976]. Overall, these data point to strict differences in tissue susceptibility and that further oncogenic signaling is required to account for malignant tumor development upon ablation of the three retinoblastoma family member genes in mouse lung.

Regarding this last possibility, we observed moderate ERK and very mild Akt activation, probably due to the induction of PTEN in tumorlets. We also observed a weak induction of Stat3, which may play oncogenic and oncosuppressive roles in lung cancer [41]. Further, we observed massive induction of p16 and p19arf in these lung lesions, whereas the pattern of p53 expression is suggestive of p53 induction rather than p53 mutation. Collectively, these findings suggest that these lesions do not progress into overt malignancy due to the induction of other tumor suppressor genes. This, together with the very low proliferation displayed by tumorlets may indicate the possible induction of senescence-like features that preclude tumor progression, probably mediated by p53 as combined inactivation of *Rb1* and *Trp53* is sufficient to cause SCLC development in mice [31, 42, 43]. This aspect could be highly relevant in the context of human lung tumor and therapies and would deserve future investigations with mouse and human patient samples.

In an attempt to provide further clarification to these possible oncogenic inputs required, we performed chemical carcinogenesis using two well known lung carcinogens, DHPN and urethane, that cause Kras activation [19, 20, 22]. We observed that under our experimental conditions DHPN is insufficient to cause tumor development in control mice, probably due to the mixed background of the strain used [44], although a potential role of p107 deficiency can not be discarded at present. In contrast, half of TKO mice developed tumors, which in this case correspond to typical carcinoids, a higher malignant grade neuroendocrine tumor than tumorlets. In the TKO mice, loss of Rb family might result in an increase in the proliferation of neuroendocrine cells favoring the progression of tumorlets to TC in the lungs of the DHPN treated group.

On the other hand, upon urethane carcinogenesis, we observed that both control and TKO mice developed tumors with similar incidence and latency. However, TKO mice displayed a higher proportion of atypical carcinoids, which are considered a more malignant grade than the typical carcinoid. Collectively, the chemical carcinogenesis experiments reinforce the resistance to overt malignant progression observed upon ablation of the three retinoblastoma proteins in lung even upon activation of oncogenic signals such as Kras (denoted by ERK activation). Nonetheless, these experiments also indicate that the possible malignant evolution of the mouse lung tumors to typical and atypical carcinoids is facilitated by Rb-family loss.

Of note, all the tumors that arose in our cohorts were of neuroendocrine type. In this case, given the absence of spontaneous tumorlets in the control untreated group, we could consider a potential role for p107 loss alone that could lead to the change to neuroendocrine cell phenotypes after urethane treatment. Urethane is known to induce lung adenomas/adenocarcinomas in A/J mice [25, 45] and the susceptibility to develop pulmonary tumors varies markedly among mice of different strains [21, 46]. Our studies, performed in mice with a triple genetic modification, were carried out in littermates of a mixed background so we cannot discard the possibility of strain related characteristics or effects due to the nullizygosity status of p107. In this regard, loss of p107 has been reported to give different phenotypes depending on the strain background [47, 48]. The tumors occurring in control mice upon urethane treatment displayed generalized loss of pRb expression (Supplementary Figure S4B). This is in agreement with the data mentioned above supporting that the loss of pRb favors neuroendocrine differentiation and tumor development. Reduced expression of *Rb1* mRNA in urethane induced tumors in different genetic backgrounds has also been reported [27] providing a further explanation for the development of NE tumors.

In summary, our findings support that the inactivation of all the three members of the Rb family in the lungs renders neuroendocrine lung tumor formation but is not enough to allow spontaneous tumors to progress into a higher state of malignancy. These data provide evidence for the link between loss of Rb family members and development of specifically neuroendocrine tumors and points out to the requirement of other signaling pathways for the malignant progression.

## MATERIALS AND METHODS

### Mice and adenoviral infections

All the animal experiments were approved by the Animal Ethical Committee and conducted in compliance with Centro de Investigaciones Energéticas, Medioambientales y Tecnologicas (CIEMAT) Guidelines. *Rb^F/F^; p130^F/F^;p107^−/−^* mice were previously described [15]. Rosa26R reporter animals [23] were purchased to The Jackson Laboratory. Ablation of *Rb1* and *Rbl2* in pulmonary cells was achieved by intratracheal or intranasal administration of adenovirus Ad5-CMVcre [29] to 8–10 week old mice. The dose of Ad5-CMVcre ranges from 5 × 10^8^ pfu to 10^10^ pfu. Ad5-CMVcre was obtained from University of Iowa's Vector Core Facility (www.uiowa.edu). As control animals, *Rb^F/F^; p130^F/F^;p107^−/−^* littermates were used. Mice were sacrificed 9 to 24 months after the Ad5-CMVcre infection.

### Carcinogen administration

A group of animals were treated with chemical carcinogens (DHPN or urethane). Chemical carcinogen was administered both to adenovirus infected mice and to control group (non-infected littermates). Groups are then referred to as TKO+DHPN; control+DHPN; TKO+ urethane; control+urethane.

DHPN (Sigma-Aldrich, Madrid, Spain) was administered in drinking water in a 0.1% concentration for 8 weeks, both to uninfected (*n* = 10) and infected mice (*n* = 12). DHPN administration began 4 or 22 weeks after Ad5-CMVcre delivery. Mice were sacrificed 35 weeks after starting DHPN treatment (Figure 4).

Urethane (Sigma-Aldrich, Madrid, Spain) was administered intraperitoneally (1 mg/g bodyweight) once a week for 10 weeks and additional 10 weeks after a month without treatment to uninfected control littermates (*n* = 11) or infected mice (*n* = 18). For the adenovirus infected mice urethane administration began 4 or 22 weeks after Ad5-CMVcre delivery. Mice were sacrificed 24 to 29 weeks after the beginning of the treatment.

### Immunostaining and X-gal staining

At necropsy, lungs were perfused with 4% formaldehyde or 70% ethanol. Samples were fixed in 4% buffered formalin or in 70% ethanol and embedded in paraffin wax. Sections (5 μm) were stained with hematoxylin and eosin (H/E), for histological analysis, or processed for immunohistochemistry. Immunohistochemical and immunofluorescence analysis were performed essentially as previously described standard protocols [49, 50]. Antibodies used were: anti-Neural cell adhesion molecule (NCAM 1, Millipore, diluted 1:200), anti-chromogranin A (CHR A, Abcam; diluted 1:400), anti-synaptophysin (SYN, Dako; diluted 1:20), anti-thyroid transcription factor-1 (TTF-1 ,Abcam-Epitomics; diluted 1:200), anti-Prosurfactant Protein C (proSP-C, Millipore, diluted 1:1000), anti-Clara cell secretory protein (T-18) (CC10, Santa Cruz Biotechnology, diluted 1:100), anti-Clara cell secretory protein (H-75) (CC10, Santa Cruz Biotechnology, diluted 1:100), anti Calcitonin gene regulated peptide (CGRP, Sigma Aldrich, diluted 1:8000), anti-aquaporin5 (Aq5, Abcam diluted 1: 200), anti-p16INK4a (Santa Cruz Biotechnology, diluted 1:100),anti-p19ARF (Abcam, diluted 1:100 anti-p53 (Novocastra, diluted 1:500), anti-Cyclin D1 (Clone SP4) (CCND1, Thermo Scientific, undiluted), anti-phospho-ERK (Cell Signaling, diluted 1:400), anti-signal transducer and activator of transcription 3 (STAT3, Cell Signaling, diluted 1:100), anti-Phospho-AKT (Ser473) (Cell Signaling Technology, diluted 1:100), anti-phosphatase and tensin homolog (PTEN, Cell Signaling, diluted 1:200), Cleaved Caspase-3 (Asp175)(CASP3, Cell Signaling, diluted 1:200), anti-Ki-67 (Clone SP6) (Thermo Scientific, undiluted), anti-phospho-Histone H3 Ser10 (PH3, Millipore, diluted 1:200), anti-pRb (Pharmingen, diluted 1:100), anti-p130 (Santa Cruz sc-317, diluted 1:200) and anti-cre (Novagen, diluted 1:1000). For inmunohistochemical analyses of human tumorlets, automatic BOND III of Leica was used. Antibodies used were: Anti-chromogranin (Novocastra-Leica, diluted 1:200), anti-CD56 (Novocastra-Leica, diluted 1:80), anti-synaptophysin (Dako, diluted 1:50), anti-TTF1 (Novocastra-Leica, diluted 1:200), anti-MIB-1 (Dako, diluted 1:100) for the Ki-67 protein, anti-p16 (Ventana-Roche, diluted 1:10), anti-PTEN (Dako, diluted 1:150).

For quantification of the number of Ki-67and PH3 positive cells, analysis of tumor areas and images was done using ImageJ software. More than 1000 cell nuclei were counted.

To detect β-galactosidase activity whole lungs were fixed in PBS containing 2% paraformaldehyde and 0.25% glutaraldehyde for 2 h at 4°C prior to incubation with X-gal solution at 37°C overnight. These tissues were then postfixed in 4% paraformaldehyde and embedded in paraffin. Sections were then processed for hematoxylin staining, immunohistochemistry or both, and examined by light microscopy.

### RT-qPCR

RNA was isolated from whole mouse lungs in control and TKO mice and from isolated tumors in urethane-treated mice using RNALater (Ambion) and miRNeasy Mini Kit (Qiagen) according to the manufacturer's instructions. Genomic DNA was eliminated from the samples by a DNase treatment (Rnase-Free Dnase Set Qiagen). The Omniscript RT kit (Qiagen) and oligo dT primers were used to prepare cDNA from RNA of the mouse samples, using 2 μg of total RNA.

RNA was obtained from isolated tumorlets embedded in paraffin using the RNeasy FFPE Kit (Qiagen) according to the manufacturer's instructions. Genomic DNA was eliminated from the samples by a DNase treatment (Rnase-Free Dnase Set Qiagen). The Omniscript RT kit (Qiagen), sequence-specific primers and random primers were used to prepare cDNA from RNA of the mouse samples, using 100 ng of total RNA.

Real-time quantitative PCR was done on a 7500 Fast Real-Time PCR system (Applied Biosystems) with the GoTaq pPCR Master Mix (Promega), using 1 μl of cDNA (as a template). Melting curves were performed to verify specificity and absence of primer dimerization. Reaction efficiency was calculated for each primer combination and each sample was normalized using the values for the TATA binding protein gene (Tbp). The sequences of the specific oligonucleotides used are listed in Supplementary Table S1. Discrimination between samples showing increased or decreased relative expression was made using the Mean ± SEM.

### Statistical analysis

Comparisons between two groups were performed using Student's unpaired *t*-test or Mann-Whitney test depending on the normal distribution of the data. The association between categorical variables was analyzed using Fisher's exact test. Statistical significance was accepted at *p* < 0.05. Graph prism 5.0 software was used.

## SUPPLEMENTARY FIGURES AND TABLE


