# Correction: Ablating all three retinoblastoma family members in mouse lung leads to neuroendocrine tumor formation

**DOI:** 10.18632/oncotarget.19510

**Published:** 2017-07-24

**Authors:** Sara Lázaro, Miriam Pérez-Crespo, Ana Belén Enguita, Pilar Hernández, Jesús Martínez-Palacio, Marta Oteo, Julien Sage, Jesús M. Paramio, Mirentxu Santos

**Present**: The grant support section is incomplete.

**Correct**: The complete grant support information appears below.

Original article: Oncotarget. 2017; 8:4373-4386. https://doi.org/10.18632/oncotarget.13875

**FUNDING**

Funds from Fondo Europeo de Desarrollo Regional (FEDER) and by grants from the Ministry of Economy and Competitiveness of Spain (AES grants ISCIII-FIS PI12/01959 and PI15/00993 to M. Santos; ISCIII-RETIC RD12/0036/0009, ISCIII PIE15/00081, MINECO grants SAF2012–34378 and SAF2015–66015-R to J.M. Paramio) and Comunidad Autónoma de Madrid grant S2010/BMD- 2470 (Oncocycle Program) to J.M. Paramio.

