# Supplementary material for: Ablating all three retinoblastoma family members in mouse lung leads to neuroendocrine tumor formation
Source: Oncotarget. 2016 Dec 10;8(3):4373–86. doi: 10.18632/oncotarget.13875 (PMC5354839; doi:10.18632/oncotarget.13875)
Supplement: Supplementary file 1 [file oncotarget-08-4373-s001.pdf]

Ablating all three retinoblastoma family members in mouse lung leads to neuroendocrine tumor formation

Supplementary Materials

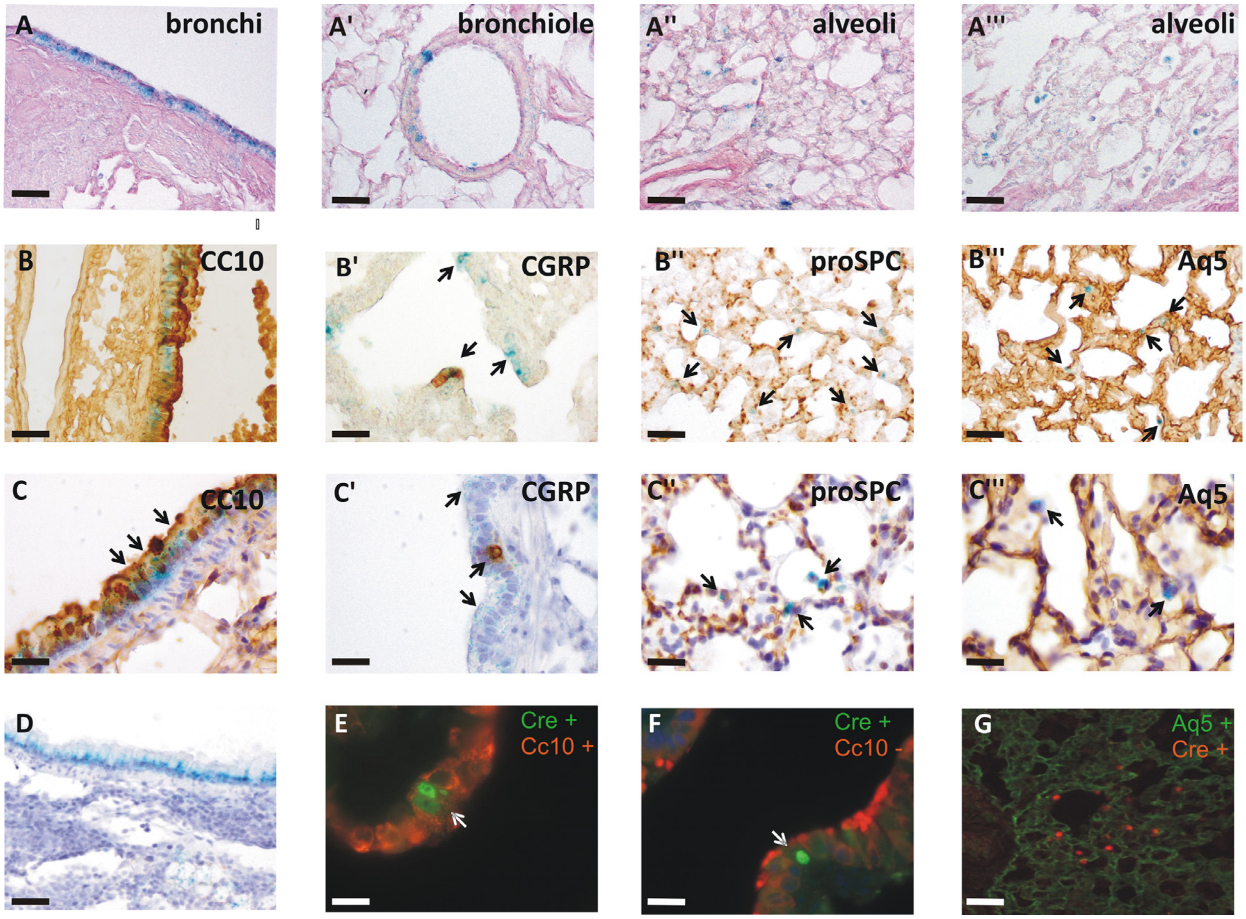

Supplementary Figure S1: Ad5CMV cre infection targets all types of epithelial cells in the adult mouse lung. (A–A´´´) Eosin and X-gal staining showing LacZ+ cells in bronchus (A), bronchiole (A´) and alveoli (A´´, A´´´) epithelial cells. Bar = 100 µm (**B–C´´´**) Immunohistochemical staining of the epithelial cell-specific markers indicated in paraffin sections of X-gal stained ROSA 26R mouse lungs 6 days after Ad5CMV cre infection. Colocalization of LacZ+ cells with anti-CC10 (Clara cells) (B, C), anti-CGRP (neuroendocrine cells) (B´,C´) , anti-pro-SPC, (alveolar type II cells) (B´´,C´´), anti-aquoporina 5, (alveolar type I cells) (B´´´, C´´´). (C–C´´´) Sections counterstained with hematoxylin. Each row (A–A´´´ Bars = 100 µm), (B–B´´´ Bars = 50 µm) and (C–C´´´ Bars = 50 µm) shows cell-specific epithelial cell markers from a different animal. (**D**) Hematoxylin and X-gal staining showing LacZ+ cells in a wide variety of epithelial cells. Bar = 100 µm (E–G) Immunofluorescence based colocalization of cre and the indicated epithelial cell-specific markers in paraffin embedded sections of TKO mouse lungs 4 days after Ad5CMV cre infection. Bars = 20 µm (**E**), 50 µm (**F**), 100 µm (**G**). (A–A´´´) dose of Ad5-CMVcre used: 1010 pfu (B–D) 5 × 10^8 pfu.

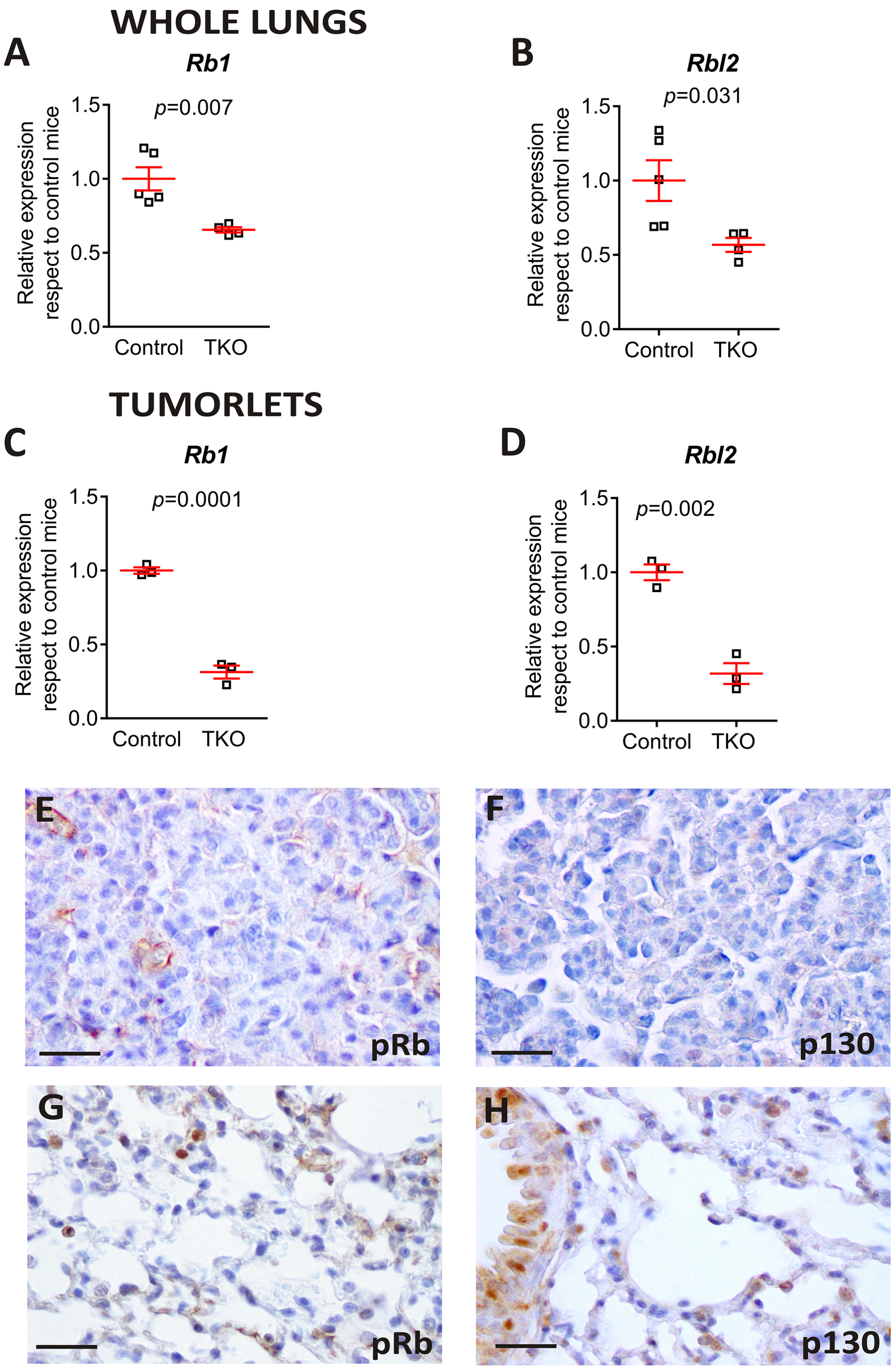

Supplementary Figure S2: Effective ablation of conditional genes (*Rb1*, *Rbl2*) in infected lungs and tumorlets. (**A**, **B**) RT-qPCR shows decreased *Rb1* and *Rbl2* in adeno-cre infected whole lungs (A, B, control, *n* = 5; TKO, *n* = 4) and in isolated tumorlets (**C**, **D** control, *n* = 3; TKO, *n* = 3). *Rb1* (A, C) and *Rbl2* (B, D) expression are significantly reduced relative to controls (Student´s unpaired *t*-test) (**E**–**H**) Representative examples (*n* = 4) of the immunohistochemistry analysis of pRb (E, G) and p130 (F,H)expression in lung and tumorlets sections from Rb$^{F/F}$; p130$^{F/F}$; p107$^{-/-}$ mice upon Adeno-cre infection. Note the absence of staining in tumorlets (E, F) compared to lung alveoli (G, H). Bars = 50 µm.

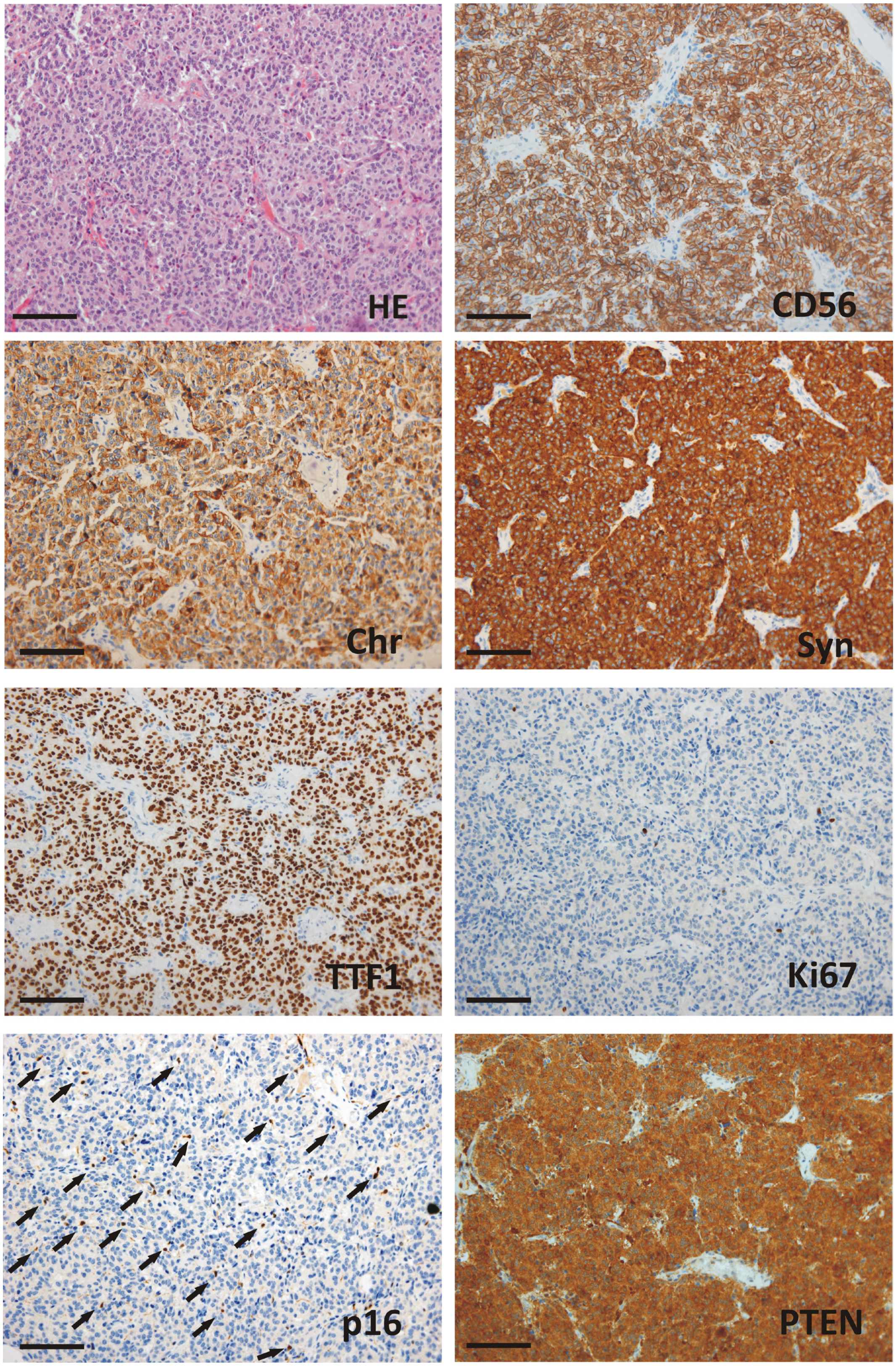

Supplementary Figure S3: Immunohistochemical analysis of human tumorlets. Histology and immunohistochemical staining of the quoted proteins. CD56, CHR, SYN, TTF-1, Ki-67, p16 (arrows) and PTEN in a human tumorlet. The quoted proteins are expressed in human tumorlets. Bars = 100 μm.

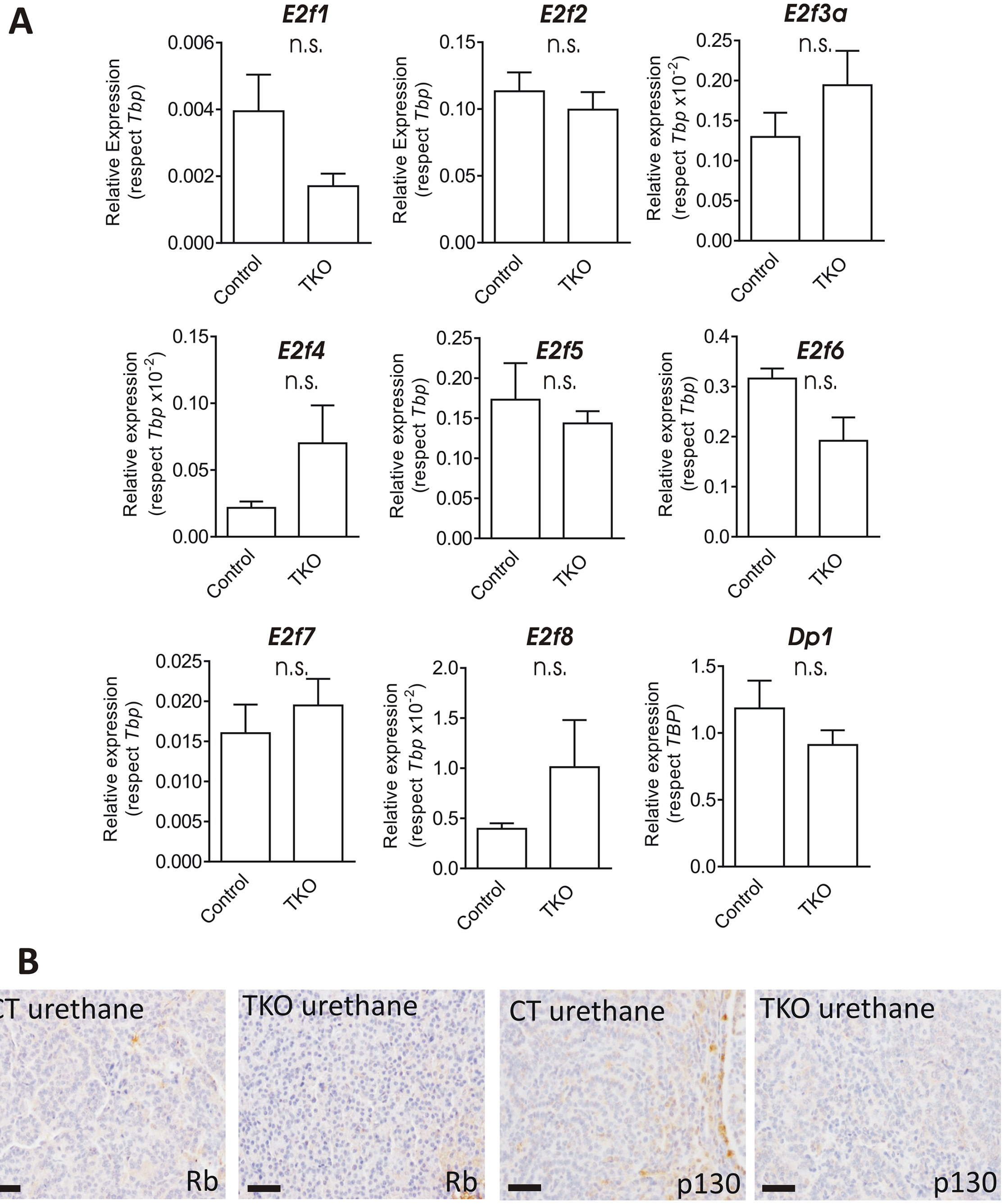

Supplementary Figure S4: Analyses of expression of E2Fs transcription factors, Rb and p130 in urethane treated mice. (**A**) Expression of *E2f* family genes in isolated tumors after urethane treatment in control (uninfected) and TKO (Adeno-cre infected) assessed by RT-qPCR (respect to Tbp). Comparisons were performed using Mann Whitney test (control n = 6, TKO n = 9. n.s = not significant). Comparisons were not significant ($p > 0.05$). (**B**) Immunohistochemistry staining of pRb and p130 in tumors from the urethane treated quoted groups. Bars = 100 µm.

Supplementary Table S1: The sequences of oligonucleotide primers used in qPCR analyses

OLIGONUCLEOTIDE	5′-3′ SEQUENCE
DP1f	GCTCAGCAGTGCCAGAACTT
DP1r	TGGAGCTGTGACTGCTTCTG
E2F1f	TGCCAAGAAGTCCAAGAATCA
E2F1r	CTTCAAGCCGCTTACCAATC
E2F2f	GGCTTCCCCAGTCTTTTTGAT
E2F2r	TGTGAGCTTGTTCCCACGCTA
E2F3af	CCCGCCCTGGAGCAGTA
E2F3ar	CCCAGTTCCAGCCTTCG
E2F4f	GAACTGGACCAGCACAAGGT
E2F4r	CATGAGTCACGTAGGCCAAGC
E2F5f	AATGATTACCTGTCCAGCAGCTG
E2F5r	GCTGTAACTGCCCTCTCAGGAG
E2F6f	GCACCAAAGGACCCATTGAT
E2F6r	GCATTGTGGAT GGCTGCTT
E2F7f	TGTTACGTGAGACATCCGGTA
E2F7r	GGATGCTCTTGGGAGTCG
E2F8f	GGCATTCAGACATGTGCTTCG
E2F8r	GCTCATCACGCGTAAGGACTT
P130f	AAGGCACATGCTAACCAATGAA
P130r	GAGCAGTTACCGCAGCATGA
RBf	CACGTGTAAATTCTGCTGCAA
RBr	ACAGGGCAAGGGAGGTAGAT
TBPf	GGGAGAATCATGGACCAGAA
TBPr	GATGGGAATTCCAGGAGTCA

Oligonucleotide sequences of the sequence-specific primers used for the RT reaction in isolated tumorlets embedded in paraffin.

OLIGONUCLEOTIDE	5′-3′ SEQUENCE
P130	ATTCTGGTTAAAGATACATA
RB	AACAACTTGCTTTCATATT
TBP	AAAATAGAGAGACTGTTGG
